# Framing openness: Exploring Similarities and Differences in Patients’ and Their Social Networks’ Experiences with Participating in Dialogical Network Meetings Through the Lens of Mattering

**DOI:** 10.1007/s10597-024-01354-8

**Published:** 2024-09-17

**Authors:** Siri Omvik, Ragnhild Andersland, Øyvind Reehorst Kalsås

**Affiliations:** 1 Western Norway University of Applied Sciences, Bergen, Norway; 2 Kronstad District Psychiatric Center, Helse Bergen HF, Bergen, Norway

**Keywords:** Social networks, Open dialogue, Network meetings, Public mental healthcare, Mattering

## Abstract

In this qualitative study conducted at a public mental health outpatient clinic in Norway, the integration of patients’ social network in treatment was examined. The aim was to explore the experiences of patients and their network during dialogical network meetings and discuss any similarities and differences between the two participant groups. Reflexive thematic analysis was performed on data obtained from fifty-three meetings, resulting in the development of five themes. For patients, there were two themes: “Enhanced trust within our relationships” and “Providing us a safe space to talk openly,” and for network members there were three: “Empowered through participation,” Being welcomed and taken seriously,” and “Provide more clarity to enhance our ability to contribute.” Mattering was employed as a conceptual framework to discuss the similarities and differences between the themes of the two participant groups. Both patients and network members placed strong emphasis on the freedom of expression and acknowledged the crucial role of the meeting leaders in facilitating discussions on important and challenging topics. Differences included network members’ emphasis on feeling welcome and their need to add value, while patients emphasized strengthened relationships and feeling valued and empowered by being trusted to control the discussion content. Overall, mattering appears to be a valuable tool for understanding the relational dynamics within network meetings.\.

## Introduction

Therapeutic approaches that systematically integrate family and social network in treating mental health issues have been linked to improvements in patient outcomes in various areas (Bergström et al., 2022; Buus et al., 2019; Stratton, 2016). Additionally, it has been shown to positively impact the situation of the patients’ relatives (Jeppesen et al., 2005) and can foster healthier dynamics within the patient’s family (Buus & McCloughen, 2022; Gidugu et al., 2021). Norwegian health authorities advocate for family participation in mental health care (Norwegian Directorate of Health, 2017). Still, actual practice falls short, with recent studies documenting infrequent implementation (Bjørkquist & Hansen, 2017; Gotaas et al., 2021; Kalsås et al., 2020).

One way to involve relatives and network members in the treatment process is through network meetings. Many mental health studies that focus on integrating social networks into treatment have primarily concentrated on Open Dialogue, which includes network meetings based on dialogical practice (Freeman et al., 2019). Open Dialogue is commonly referred to as a comprehensive therapeutic framework that emphasizes immediate response and placing the social network at the center of therapeutic efforts throughout the entire mental health service provision (see Buus et al., 2021; Ong et al., 2019; Seikkula et al., 2011; Seikkula & Arnkil, 2006).

The Open Dialogue principles include guidelines for facilitating network meetings in adherence to dialogical practice in Open Dialogue. These guidelines, as outlined by Olson et al. (2014), encompass twelve key elements that should permeate the facilitation of the network meetings. Some central elements of these guidelines include the presence of two or more therapists in the meeting, the use of open-ended questions, active responses to patients’ utterances, reflection among professionals during the meetings, and promoting transparency and tolerance for uncertainty (Olson et al., 2014). By following these guidelines, mental health systems with less flexibility than an Open Dialogue-based system can potentially integrate dialogical network meetings into their treatment processes, thus incorporating elements of Open Dialogue within their existing structures. For instance, systematic offering of dialogical network meetings in a closed psychiatric ward has been reported to yield positive results for both patients and network members (Sørgård & Karlsson, 2017).

One of the findings in a review of research on Open Dialogue was that more qualitative research is needed on how service users experience network meetings (Freeman et al., 2019). Additionally, the review emphasized the importance of comparing the perceptions of individuals with different roles in these meetings. Subsequent qualitative studies have predominantly focused on exploring the collective perceptions of patients and network members. These studies often center around the experiences related to piloting Open Dialogue as a comprehensive approach to care, which includes network meetings but is not limited to them (e.g. Florence et al., 2021; Gidugu et al., 2021; Hendy & Pearson, 2020; Wusinich et al., 2020). Most findings from these studies show positive experiences and changes from participating in a mental health system based on Open Dialogue approach and network meetings. These experiences include feelings of mutuality with the professionals’ facilitating the meeting (Hendy & Pearson, 2020), changes in self-understanding, understanding of others, network relationships, and their dynamics (Wusinich et al., 2020), reduced stigma (Florence et al., 2021), and strengthening of relationships (Buus & McCloughen, 2022).

Several studies also shed light on challenges associated with conducting and participating in dialogical network meetings. A study involving 17 persons receiving services based on Open Dialogue in Vermont reported that participants sometimes experienced difficult tensions and unsafe atmosphere in the meetings (Florence et al., 2021). Similarly, an Australian study involving four patients and 14 network members, reported that participants found themselves engaged in complex practical, emotional, and interpretative work during the meetings (Buus & McCloughen, 2022). Moreover, a study in the UK, which included five service users and three network members, emphasized that some service users felt unsure about the purpose and expectations of the meetings, felt uncomfortable and unsafe when emotional expressions were shared too openly, and questioned the genuineness of interactions with professionals (Tribe et al., 2019).

While the mentioned studies primarily rely on joint analyses of patient and network members’ statements, certain studies incorporate illustrative quotes from the social network. For instance, a study conducted in Massachusetts, the theme of family involvement was illustrated through a family member who described a newfound ability to support the patient in a manner he could accept, leading to a stronger relationship between them (Gidugu et al., 2021). The experience illustrates that the position and role of a network member in many ways can be different from that of a patient. Thus, it is likely that their needs and experiences concerning participating in social network meetings differ in some ways and are similar in others. Still, there is a scarcity of studies illuminating similarities and differences between patients and network members in participating in network meetings.

In the current study, we specifically aimed to compare the experiences of participants in separate roles in these meetings, focusing on patients and network members in a public mental health setting in Norway. The research question posed was: “What positive aspects, and what proposals for change, do patients and their social network highlight after attending one or two network meetings at a mental health outpatient clinic?” Furthermore, upon analyzing the data, it became evident that the concept of mattering as outlined by Prilleltensky (2020), provided a relevant framework for enlightening similarities and differences in the themes between the two participant groups. Accordingly, we also aimed to discuss the findings through this lens.

## Methodology

### Design

This qualitative study analyzes written statements from patients and network members about their experiences with dialogical network meetings, using a hermeneutical approach. We used reflexive thematic analysis to interpret patterns in the statements. The study is part of a broader research project examining the influence of these meetings on patients’ mental health, which includes a quantitative treatment study measuring the impact on various outcomes (see Omvik & Kvamme, 2022).

### Context

The study setting is a public mental health hospital in Bergen, a city on the west coast of Norway with about 300,000 residents. The hospital’s catchment area spans from the city center to its outskirts, offering voluntary mental health services to approximately 90,000 adults within this region. It provides specialized services and accommodates both inpatient and outpatient wards.

The study was conducted within a group unit at the hospital, where network meetings were introduced as an additional option for patients participating in a group-based day treatment program. The program entailed four weeks of treatment three days a week, encompassing multiple components, including psychoeducation, body awareness, physical activity, group therapy, and shared meals. An interdisciplinary team comprising psychiatric nurses, a psychologist, a physiotherapist, an occupational therapist, a peer support worker, a social worker, and a psychiatrist managed the program, accommodating eight patients at a time. Each patient was assigned a therapist responsible for their treatment, with whom they had preparatory and concluding sessions and could seek support as needed. The program’s primary objective at the time of the study was to assist patients in improving their coping strategies and foster hope for recovery. Most participants were referred internally from other hospital units, and the program aimed to help the transition phase after discharge from the inpatient ward or, in cases where weekly outpatient follow-up was insufficient, act as an alternative to inpatient treatment. The patients typically experienced affective, anxiety, personality, or trauma disorders. Suicidal thoughts and behaviors were common, while having a primary psychotic disorder was an exclusion criterion.

#### Network Meetings in the Treatment Program

Before the current study, dialogical network meetings were offered non-systematically to some patients in the day program by a staff member who was formally trained in this way of working. Based on positive feedback, we decided to extend the offer to all patients in the program and assess the usefulness of the meetings. Thus, the network meetings were introduced as an optional element that patients could accept or decline. Due to the program’s brief duration, we limited the number of meetings to a maximum of two per patient.

The meetings were guided by the Open Dialogue principles of dialogism and tolerance of uncertainty (Olson et al., 2014) and were facilitated by therapists trained in dialogical practice. All meeting leaders worked in the day program except one. Participants in the meetings included family members and other members of the social network. There was no pre-set agenda, and all participants were encouraged to share their own experiences and thoughts regarding the current situation. Open-ended questions were posed to elicit a variety of perspectives. The meeting leaders aimed to create an informal and friendly atmosphere and strived to use the same language as the patient and network members to reduce distance and avoid taking an expert role. At times, they talked together about their reflections while the participants listened.

The training in dialogical practice was provided by experienced network meeting leaders. It lasted approximately six months and included reading and discussing literature, watching videos of network meetings and discussing them, and participating in role plays to practice leading meetings. The therapists also participated in one or two real network meetings together with an experienced leader before they began leading meetings themselves. Additionally, they attended monthly group supervision sessions during the project, where they discussed issues from the network meetings concerning dialogical principles to ensure safe dialogical practice.

Meeting leaders’ adherence to dialogical principles were assessed using a self-developed Likert scale. The scale contained eight questions aimed at dialogical aspects of the meeting, such as openness, interest, and respect, and could be answered on a scale ranging from 0 (“not at all”) to 4 (“to a very large extent”). Both patients and network members completed the scale after each meeting. The average for each question indicated that the principles “to a large extent” were adhered to, implying that the fidelity was satisfactory. More detailed results can be found in the quantitative study by Omvik and Kvamme (2022).

Patients who agreed to arrange network meetings first met with their therapist to discuss the details, such as location and time. Subsequently, the patients themselves invited members from their network whom they wished to involve. The meetings typically lasted 60 to 90 min and were led by two meeting leaders, one of whom was the patient’s therapist. Although the meetings could be held at various locations, all participants chose to have them at the hospital. After the initial network meeting, patients could schedule another one. Half of them arranged a single meeting, while the others organized two. The composition of participants varied, with some patients inviting the same people twice, others inviting only new people, and some a combination of both.

### Data Collection Process

All patients referred to the day program between August 2017 and May 2019 were invited to participate in the study. Aim and procedures were explained during the individual preparatory consultation with their therapist. Regardless of their decision concerning participation, all patients were subjected to the same assessment procedures. However, only those who agreed to participate signed an informed consent form granting permission for their data to be included in the study. The consent form covered both the quantitative and qualitative study in the project. Participation in the day program was not conditional on the patients’ consent. The network members were invited to participate in the study after attending the meetings. At the beginning of each meeting, the leaders briefly introduced the study and its purpose, informing them that more detailed information would be provided at the end. Once the meetings concluded, members who expressed interest were presented with an informed consent form that outlined the study. Those who wanted to participate read and signed the form. Thereafter, questionnaires were distributed to network members and patients, who completed them in the same room where the meeting had taken place. The procedures allowed everyone to see who consented to participate. The responses from both network members and patients were collected anonymously in sealed envelopes. Each envelope was marked with an ID number that could be linked to the patient. The consent forms were assigned the same ID number and kept separately from the questionnaires. On the first page of the questionnaire, participants were asked to indicate their role in the meeting (e.g., patient, parent, sibling, friend, etc.). In conjunction with the ID number, this information made it possible to match network members to their responses, enabling the deletion of their information upon request.

### Study Sample

#### Patients

**Fig. 1:**
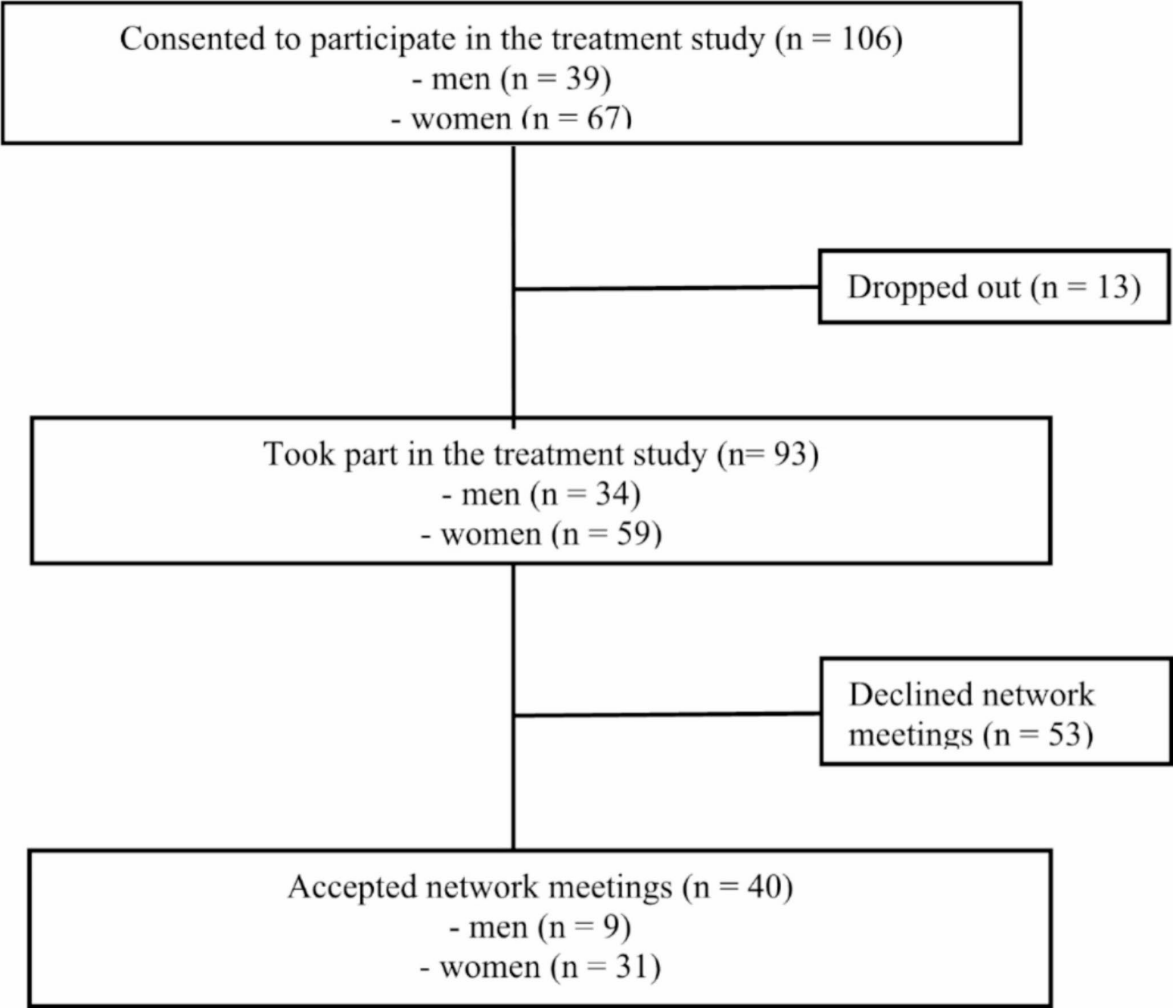
Recruitment of patients for the treatment study and the current qualitative study

A total of 40 out of 93 patients (43%) who participated in the treatment study agreed to arrange network meetings (see Fig. 1 for an overview of the recruitment process). Among them, 9 (22.5%) were men and 31 (77.5%) women, and their ages ranged from 19 to 68 with an average of 34.6 years. Regarding work status, 42.5% reported receiving a disability pension, work clarification allowance, or unemployment, while 57.5% reported being employees or students. All participants reported significant impairments in daily functioning, such as being unable to work or study, or experiencing difficulties maintaining close relationships. Additionally, nearly all scored above the clinical cut-off for depression and anxiety (Omvik & Kvamme, 2022).

#### Network Members

For each meeting, we recorded the number of participants from the patient’s network and their relationship with the patient. To ensure anonymity, no additional information was collected. The identities and counts of those not participating in the study were not recorded. As a result, the actual number of participants may have been slightly higher than reported here at some of the meetings.

A typical network meeting involved four people: the patient, two meeting leaders, and a parent or partner of the patient. This composition was observed in approximately 35 of the 60 meetings arranged. In 15 of the meetings, there were two participants from the patient’s network, while in 10 meetings there were three or more. Parents were the most frequent participants, present in over half of the meetings, followed by partners who attended about a third. Siblings, friends, and children were also registered as participants.

### Data Material

We aimed to collect the participants’ immediate and honest experiences of the meetings, and we wanted to ask both open-ended questions about their experience with participating and quantitative questions addressing adherence. To achieve this, we used anonymous questionnaires that were answered immediately after the meetings. Participants were asked to rate the meetings on selected dimensions in the quantitative items. In the open-ended qualitative questions, they were asked to provide more detailed feedback on their experiences. This study reports findings from the open-ended questions that were stated as follows:

- “Was there anything that could have been different during the network meeting? If so, what was it?”
- “Was there anything about the meeting that you felt were good for you? If so, what was it?”
- “Any other comments?”

A total of 60 network meetings were organized, as half of the participants arranged two meetings. At least one participant answered the open-ended questions in 53 of these. All responses were used as data, and the complete data material comprised around 200 responses. These varied in length and content, ranging from shorter statements to longer sentences of up to three lines, which covered several topics. The most common response consisted of about one line of text. Of the 40 patients who arranged network meetings, 38 were represented in the material either through their own answers and/or through answers from their network members. As the analysis progressed, statements that were assessed as irrelevant to the research question in terms of being too general or not in accordance with the developed themes were excluded. The final analysis includes 50 text segments from patients and 92 from network members from 46 different network meetings.

### Data Analysis

The three authors of this article were all involved the analysis of the data. S.O., a psychologist, served as the professional responsible for the day treatment program and as the study’s project leader during data collection. However, she was not a therapist in the program or at the network meetings. R.A., an occupational therapist, was employed in the day treatment program when the meetings were implemented and participated as a meeting leader in some of the network meetings in the project. Ø.R.K., a social worker with expertise in leading network meetings and knowledge of qualitative method, was never employed at the treatment center and did not participate in the network meetings or data collection. While R.A. represented a very close position to the data analysis since she had attended some of the meetings, S.O. provided a more distant perspective based on the involvement in the comparative quantitative study (Omvik & Kvamme, 2022), and Ø.R.K. represented a position outside the treatment context but with relevant expertise.

#### Reflexive Thematic Analysis

Reflexive thematic analysis offers flexibility for both descriptive and interpretative analyses (Braun & Clarke, 2022). The data consisted of short written answers, and we considered this flexibility potentially useful when approaching the data. Braun and Clarke (2022) outline six phases in thematic analysis: (1) familiarization with the data, (2) coding, (3) generating initial themes, (4) developing and reviewing themes, (5) refining, defining and naming themes, and (6) writing up. The authors recommend moving back and forth between phases 2 and 5 before writing up. Our analysis followed these procedures and was conducted using NVivo, version 14 (Lumivero, 2023) together with manual review of the material. Responses from patients and network members were analyzed separately. We segmented responses into smaller units if they contained multiple meaningful entities.

Before starting the analysis, all authors engaged in reflexivity, documenting and sharing experiences and positions as clinicians and professionals regarding dialogical network meetings and expectations regarding the data. In the first phase of the analysis, R.A. familiarized herself with the dataset and presented initial ideas for the analysis. Subsequently, S.O and Ø.R.K also familiarized themselves with the material by reviewing it multiple times, taking notes, and recording initial ideas and potential codes.

In the coding phase, S.O. created codes and themes based on shared ideas and reflections. These were discussed and modified at analysis meetings in five rounds before the results and discussion were written up. The process was also repeated in a sixth and seventh round, based on feedback from reviewers after submission to this journal. The coding process followed an inductive and semantic approach, at first closely aligning with the semantic expressions in the data. As the analysis unfolded, coding labels were developed from interpretations of more latent meanings that evolved from discussions and the iterative movement between the whole and the parts during the thematic analysis phases. Interpretations were influenced by the researchers’ diverse backgrounds and varying relationships with the data. R.A., who actively participated in the meetings, witnessed significant emotional exchanges between patients and their network members, and recognized the importance of integrating patients’ networks to a greater extent in mental healthcare. She played a significant role in recognizing the vulnerabilities of both patients and network members. S.O., on the other hand, had initially doubted whether the network meetings would lead to improvement beyond the help the patients received in the day treatment program. However, these doubts were reduced after the quantitative study showed that carrying out network meetings was associated with reduced symptoms. During the qualitative analysis, S.O. was attentive to how the meetings could contribute to changes in the patients’ mental state. Finally, Ø.R.K.’s experience with dialogical network meetings in other contexts proved valuable in generating deeper interpretations and recognizing phenomena known in Open Dialogue, such as the significance of sharing emotional topics with one another. The perspectives complemented each other, contributing to a comprehensive understanding of the study’s context and enriching the discussion.

An example of the evolving interpretation and reflexive process can be seen in how we understood that two of our generated patient themes from the early rounds, specifically “Being able to talk about what I need” and “Professional frames for the dialogue”, concerned the same phenomenon. In the group discussions, it was S.O. who particularly emphasized the significance of the meeting leaders’ role in establishing a safe space for important dialogue about difficult topics. This emphasis aligns with her background as a psychologist and understanding the importance of the therapeutic relationship in treatment. Eventually, we arrived at a broader theme encompassing both previous themes, “Providing us a safe space to talk openly.”

In the last phase of the analysis, we evaluated whether the final themes met the requirements for encapsulating central organizing concepts related to the data and the research question (see Braun & Clark, 2022), and found this satisfactory.

### Ethical Considerations

Prior to commencing the study, it was presented to the Regional Ethics Committee for assessment. The committee classified it as a quality assurance project and concluded that no further evaluation was necessary. Approval to conduct the study was subsequently obtained from the health region’s data protection officer (reference number 2017/6136).

All participants, both patients and network members, received verbal and written information about the study. They were also informed of their right to withdraw at any time without any consequences for their treatment, and they provided informed consent to participate. All data were anonymized and securely stored in compliance with the current regulations on the health region’s quality server.

## Results

We generated five themes from the data, two pertaining to the patients and three to the network members (see Table 1). For the patients, both themes revolve around positive aspects they emphasize after taking part in the meetings. For the network members, the first two themes presented encompass positive aspects and the last proposals for changes to the organization of the meetings. The themes are exemplified by coded text segments, all of which have been translated into English by S.O. To illustrate the diversity of participants from which the examples are drawn, an ID number is provided for the patients’ text segments. Network members are presented with their relationship with the patient (e.g. parent, friend, etc.) and the ID number to the patient they belong to.

**Table 1:** The research question, participant roles, theme names, and theme descriptions

What positive aspects, and what proposals for change, do patients and their social network highlight after attending one or two network meetings at a mental health outpatient clinic?		
Participant roles	Theme names	Theme descriptions
Patients	Enhanced trust within our relationships	We gained a better understanding of each other’s experiences. I feel that the openness enhanced the trust within our relationships, and I became more confident that my network can support me.
	Providing us a safe space to talk openly	Letting us decide what to discuss enabled us to bring up difficult topics we needed to address. The meeting leaders were crucial in creating the safe space we needed.
Network members	Empowered through participation	Expressing our thoughts and feelings helped us feel that our voice mattered. We also gained more insight into the situation. This, in turn, made us feel better equipped to support the patient.
	Being welcomed and taken seriously	The meeting leaders facilitated dialogue about challenging topics through their non-judgmental and welcoming approach. We prefer them to be open and direct rather than protective, though.
	Provide more clarity to enhance our ability to contribute	Improving the clarity of information regarding objectives and providing more structure and concrete advice would enhance our ability to contribute effectively to the meetings and in the patients’ life beyond the treatment setting.

### Patient Themes

#### Enhanced Trust within our Relationships

The theme revolves around the patients’ experiences with engaging in dialogue with their close ones during the meetings, reflecting that they perceived an improvement in their feelings of connectedness to them. As one patient observed: “It really helped with our relationship. I feel that things got much better between us” [ID 29]. Another patient emphasized the mutual process of enhancement, stating: “[I] felt that it gave them [the network] something, which again was good for me” [ID 25]. Patients also expressed that they valued addressing challenging topics, indicating that the meetings played a pivotal role in nurturing trust. One patient remarked: “There was much openness regarding difficult matters, which was good” [ID 5]. Moreover, the theme reflects that the interactions during meetings fostered a deeper understanding, enabling patients to articulate their thoughts and gain more insight into their network members’ emotions. The benefits of this open communication were described by a patient who recounted the advantages of participating, expressing: “[I] got to put things into words and explain. I also got an insight into how those around me feel. [I became] more aware of things” [ID 17].

The theme further involves the patients’ experiences around the importance of being able to state their needs to those closest to them. Several responses show that patients felt a renewed sense of confidence in their social network’s capacity and willingness to support them. This enhanced trust is captured in statements about the positives about participating in meetings, such as: “[To] give my network a better understanding of how I feel, and how they can handle it” [ID 18], alongside the empowering realization of being able to seek support: “[That] I had the opportunity, and managed, to ask my partner for help” [ID 13]. Moreover, some patients highlighted the benefits of talking about how to deal with potential future problems, conveying an underlying sense of being valuable to their network and an expectation of receiving enduring support from them. One patient’s reflection underscores this sentiment: “[I] got to put into words how to deal with relapse together with close family” [ID 31].

In summary the theme conveys an experience among patients that the openness in the meetings contributed to a strengthening of the relational bonds and their trust in the network’s ability to provide support.

#### Providing us a Safe Space to Talk Openly

The second theme captures how patients perceived the meeting leaders as key to fostering a space conducive to constructive dialogue. By meeting them without a pre-set agenda and letting them and their network decide what topics to discuss, patients felt that their personal experiences were validated and valued. One patient acknowledged this empowering aspect, stating: “We got to control it” [ID 31]. Another highlighted the benefit of unrestrained expression, sharing: “I had the opportunity to vent my thoughts and feelings about my situation, and was met in a good way” [ID 5]. The theme also reflects the perceived significance for the network as a whole, as evidenced by one patient’s praise for how the meeting was conducted: “[A] fine execution of what we wanted and needed to talk about” [ID 17].

The theme further encompasses that the patients felt more able to discuss difficult subjects during the meetings, an ability they attributed to the facilitation by the meeting leaders. One patient expressed the impact of this supportive environment by stating: “I could discuss things that I could not talk about alone at home” [ID 7] whereas another valued the constructive nature of the discussions remarking that: “We could talk together without it becoming negative” [ID 24]. The leaders’ crucial role in establishing the necessary safety was also highlighted, for example expressed by one who described a meeting as: “A safe place to talk when you have a third party in the dialogue” [ID 12]. In addition, patients identified particular skills of the leaders that they believed improved the dialogue, such as navigating challenging subjects and inquiring with direct, clarifying questions. They also praised them for their ability to engage openly and thoughtfully with the personal and complex issues that were shared. Illustrating the value of this skilled facilitation, one patient stated: “When I explained the chaos in my head, they [the meeting leaders] helped me put it into words so that I understood it better” [ID 26].

Taken together, the theme conveys a perceived connection between the open form facilitated by the meeting leaders, and the patients’ and their networks’ ability to engage in constructive dialogue about difficult topics.

### Network Member Themes

#### Empowered Through Participation

The initial theme developed from the network members’ responses center around the significance they placed on participating and assuming an active role during the meetings, and on sharing their personal experiences. It reflects that they valued openly expressing their thoughts and feelings about the situation which contributed to a feeling that their voice mattered. One member expressed this appreciation by commenting: “It was very good that I could talk about how I experience the disease” [ID 10, friend]. Likewise, the benefit of honest expression was pointed out by another member, who mentioned: “[It was] useful for me since it made it easier to talk about how I really feel about the situation I am in” [ID 1, sister]. Furthermore, network members also emphasized the importance of their insights being an integral and eligible part of the overall dialogue, as summarized by one person’s comment: “[I] felt that my thoughts and feelings had a place and were accepted” [ID 4, sister].

In addition to reinforcing their role in the situation by taking part, network members also valued the deeper understanding they gained regarding the patients’ circumstances, including their treatment and needs. This broader perspective contributed to enhancing their ability to provide support. A mother reflected on this growth, stating: “My understanding increased. I got a much-needed wake-up call” [ID 2, mother]. Others reported acquiring a more informed stance on the healthcare system, noting for example “It was very good to learn about the test results, both past and present, from a professional point of view. It provided a sense of security” [ID 33, family member]. Several members specifically emphasized the significance of comprehending the patient’s personal situation. One mother articulated: “It became clear to me what my daughter is going through, what she experiences, and what she thinks” [ID 25]. This enhanced understanding resonated with many as contributing to improving their ability to help the patient, captured by one expressing: “As a mother, I gained a better understanding of how I can support my daughter in a positive way” [ID 28].

Taken together, the theme conveys that network members experienced a sense of empowerment in their role as providers of support to the patients by participating in the meetings.

#### Being Welcomed and Taken Seriously

The second theme for the network members pertains to their perception of the meeting leaders’ role in fostering significant dialogue within the network. Key aspects include their non-judgmental and welcoming approach towards presented issues and their genuine concern for the participants in the meetings. For instance, one network member captured the experience of participating by stating: “I feel safe in this room. [The meeting leaders are] great people who want the best for me” [ID 26, parent], while another noted that: “It was good that the topics raised were taken seriously” [ID 8, friend]. Network members also valued the space and support to engage in discourse on sensitive issues, which helped them feel more comfortable expressing troublesome thoughts. One member reflected: “[It was an] opportunity to speak openly, with support. It becomes less scary to say difficult things” [ID 5, partner]. Several conveyed an appreciation for neutral meeting leaders creating a collaborative and non-confrontational atmosphere for dialogue on sensitive issues. One member highlighted this aspect, reflecting: “I felt that I could bring up difficult topics on a neutral ground” [ID 26, sibling] while another observed: “It is the first time we didn’t quarrel” [ID 20, parent].

Although the meeting leaders were mostly praised for their validating role, a few network members mentioned instances where they felt they were overly cautious or protective in their approach. One member wrote: “There may be a slight degree of caution in the tone of voice or body language that may give the impression of overprotection. It is hard to describe exactly, but a bit unnatural” [ID 25, parent], while another stated: “At the beginning of the meeting, there was much use of “therapy voice.” While this is likely intended to be neutral, I personally found it infantilizing” [ID 10, child].

In summary, the theme revolves around the network members’ perception of the significance of the meeting leaders being non-judgmental and taking them seriously in order to manage to talk about challenging topics during the meetings. It also reflects a preference for open and direct communication rather than protection.

#### Provide more Clarity to Enhance our Ability to Contribute

The final theme for network members concerns proposals for changes in the organization and execution of the network meetings. The theme revolves around the members’ unmet needs for information and guidance and reflects that they felt uncertainty and anxiety about attending the meetings. For instance, one member wrote: “Information in advance came from the patient only, so I didn’t know what I was going to take part in” [ID 5, partner], whereas another stated: “I didn’t know what a network meeting was. I dreaded it beforehand as I thought it was the same as a confrontation meeting” [ID 26, sibling]. In addition to experiencing anxiety related to the unfamiliar situation and the unclear purpose of the meetings, network members also expressed feelings of uncertainty regarding interactions with professionals within the treatment system. One member wrote: “[I] was uncertain at first about what the meeting entailed and the extent of information they possessed about my child” [ID 17, parent]. Collectively, the statements suggest a more profound fear of being unable to handle the meeting situation.

In light of these concerns, the amendments proposed by network members can be seen as aimed at alleviating their uncertainty by establishing means to achieve clarity, control, and predictability. Among the proposals is the call for more transparent communication regarding the agenda and expectations before the network meetings. A parent underscored this need by sharing: “[I/we] could have been more prepared for what would be discussed at the meeting” [ID 25, parent]. In addition to the desire for clearer information in advance, some appealed for a more structured and focused conversation during meetings. This was articulated by a member who recommended the adoption of: “[A] meeting schedule and a written plan for the next meeting” [ID15, parent]. Moreover, some network members suggested that meeting leaders could offer more targeted reflections and specific guidance to promote greater confidence in addressing current and future challenges. One individual proposed: “Perhaps some more concrete examples of how I, as a friend, can contribute to improving the patient’s everyday life” [ID 30, friend].

Taken together, the theme presents a need for predictability and guidance among network members so that they can contribute meaningfully at the meetings and in the patient’s life beyond the treatment setting.

## Discussion

In line with previous studies, both patients and network members in our study welcomed the network meetings (see Buus et al., 2021; Freeman et al., 2019) and described improvements in the quality of their relationships (see Buus & McCloughen, 2022). They also reported an expansion of their understanding (see Wusinich et al., 2020) and an improvement in the quality of their communication (see Twamley et al., 2020). Furthermore, network members emphasized the importance of gaining insight into how they could provide support (see Gidugu et al., 2021). However, they also reported feelings of uncertainty (see Florence et al., 2021), which seemed to be related to having limited insight into the purpose of the meetings (see Tribe et al., 2019). 

While the referred studies above include findings from joint analyses from different participant perspectives (e.g., Gidugu et al., 2021; Twamley et al., 2020; Wusinich et al., 2020; Florence et al., 2021), we, in our study, conducted separate analyzes for each participant role, which allowed for a comparison of each group’s unique experiences within the network meetings. In the discussion, we focus on identifying commonalities and variations among the separate roles, illuminated through Prilleltensky’s (2020) concept of mattering. According to this framework, mattering refers to the two fundamental psychological needs of feeling valued and adding value, which are both pivotal for fostering psychological fulfilment and establishing social bonds. While the need to feel valued concerns the necessity for an individual to experience a sense of belonging, to feel valued by others, and to accept oneself and experience self-worth, adding value involves one’s ability to contribute significantly to the lives of others as well as to one’s own personal development (Prilleltensky, 2020). Feeling valued and adding value are interdependent processes. When individuals feel valued, they are more likely to be empowered to add value, and vice versa. Thus, the processes are particularly relevant in network meetings, where participants come together to engage in dialogue and support each other.

### The Impact of the Network Meetings on the Relationships within the Network

The themes developed from both the patients and the network members reflect a common emphasis on the relational benefits of talking openly about challenging topics. Articulating and receiving different perspectives emerged as important aspects that promoted mutual understanding in both participant groups, in line with the dialogic perspective on which Open Dialogue is founded (see Ong et al., 2019). Nevertheless, there are nuances in their thematic reflections regarding the emphasis on feeling understood, and on the actual act of participation.

Concerning the first aspect, feeling understood, our findings show that patients particularly emphasized how the dialogue helped to strengthen ties and trust in their social network by making them feel genuinely heard and understood by the members (see Gidugu et al., 2021; Sunthararajah et al., 2022). Framed by the concept of mattering, it thus seems that the network members’ attentive participation and increased understanding contributed to reinforce the patients’ sense of being valued, and that this sense of being valued further augmented their belief in their inherent worth to the group.

Conversely, network members focused less on being understood and more on actively engaging in the meetings by articulating their own experiences and viewpoints. While the mere invitation to participate served as an initial form of acknowledgment for them, actively sharing their perspectives seemed the most crucial validation, reinforcing their role as significant supporters to the patient. From the perspective of mattering, the active engagement can be seen as a means of affirming their self-worth, empowering them, and enhancing their sense of being able to add value.

Thus, it is an overall impression from our findings that, in terms of mattering, feeling valued was particularly important to the patients, whereas adding value was particularly important to the network members. However, a pivotal point of the conceptual framework is that both these processes are necessary for psychological well-being (Prilleltensky, 2020). This was also evident in our themes. For instance, patients articulated gratitude not only for feeling valued through the understanding they received, but also for gaining greater insight into the network members’ perspectives. Similarly, network members felt valued by receiving space and recognition for their roles, which in turn was crucial for them to feel able to add value to the patients’ lives. In sum, the experiences of feeling valued and adding value appeared to interchangeably enrich the network meetings, underlining the conceptual framework which describes these as interdependent and complementary processes.

### Implications for the Meeting Leader role

The meeting leaders in dialogical network meetings strive to foster a setting that invites a plurality of perspectives and emotions, acknowledging the diversity of individual stories (see Ong et al., 2019). Our findings underscore the vital role of meeting leaders in this endeavor. Both patients and network members particularly noted the leaders’ indispensable contribution in facilitating discussions on emotionally challenging topics and in framing the open form, which allowed participants to raise the topics they needed to discuss.

However, despite a shared recognition of the value of the open form of the meetings, our analysis points to a divergence in what this openness may represent for each participant group. For patients, the open form appears particularly important as it allowed them and their network to control the content of the discussion. Viewed through the lens of mattering, this indicates that for them, the meeting leaders’ trust in their ability to identify and raise important issues was crucial, fostering a sense of empowerment.

Contrary, for the network members, the leaders’ warm, welcoming, and open-minded approach, along with taking their contributions seriously, stood out as the most important aspect. This aligns with research showing that family members of individuals with mental health issues are often marginalized in the healthcare system (see Doody et al., 2017). Therefore, their experience of being included likely diverged significantly from their previous encounters. From a mattering perspective, this implies that when network members feel welcomed and are met in a non-judgmental way, it conveys a sense of being valued that can be transformative, affirming their role and active participation in the patient’s life and recovery. However, while the warm welcome was mostly appreciated, a few members also sensed a subtle caution among the meeting leaders, which was experienced somewhat negatively. Such wariness could unintentionally be taken as an underappreciation of their ability to handle the situation, thereby diminishing their perceived capacity to add value. Together, these finding suggest that both patients and network members have an underlying need for validation of their abilities and resources. This insight underlines the significance of conveying trust in participants through transparent and direct communication to elicit their potential to contribute meaningfully in the meetings.

The most notable contrast observed between the two participant groups, though, was that suggestions for changes to the meetings and how they should be conducted, came almost exclusively from network members. These proposals primarily focused on enhancing the predictability of the meetings, likely stemming from an apprehension about participating. From the perspective of mattering, not fully recognizing the needs of the network members at this point can be viewed as undermining their importance and ability to add value. Therefore, it appears crucial to establish transparent rules for the meetings to ensure sufficient assurance and enhance the network members’ ability to contribute effectively, especially when the context is unfamiliar to them. In this regard, the dissemination of pertinent information by leaders prior to and during the commencement of the meetings becomes central, as it can empower network members from the very start.

A final aspect that differed between the groups, was a desire among network members for receiving more guidance and advice from meeting leaders on how to address future problems which was not mirrored in the patient group. The difference suggests that network members need for more support and guidance in order to feel confident in their role, especially if the offer of network meetings is limited as it was in our study. In the context of mattering, not providing specific guidance may be perceived as a lack of validation of the significance and complexity of the network members’ role. These findings therefore indicate that the meeting leaders need to focus on, rehearse, and calibrate the balance between an open, reflective, and dialogical stance while still offering their viewpoints and professional expertise and guidance. This understanding aligns with earlier findings and discussions in the literature regarding the therapeutic challenges in Open Dialogue practices (see Buus et al., 2021).

### Implications for the Implementation of Dialogical Network Meetings in Clinical Practice

Our findings imply that the open form of the network meetings can be highly beneficial for both patients and network participants, by fostering a sense of being valued and thus becoming empowered to add value in dealing with serious mental health problems. However, it is crucial to tailor the framing of the openness to the specific treatment context and individual needs of the different participant groups involved. In line with our findings, network members may require more information beforehand, a more predictable meeting structure, and more concrete advice than patients, especially when the context and form are unfamiliar to them.

Moreover, employing mattering as a conceptual framework to interpret the dynamics of dialogical network meetings proves to be a valuable tool. Viewing the processes in meetings through the lens of adding value and feeling valued aligns with the principles of Open Dialogue (see Olson et al., 2014), and helps understand how to empower both the network and the patients. The perspective underscores the importance of establishing frames for dialogical practice to provide patients and their social network opportunities to engage in the crucial recovery processes of feeling valued and adding value.

### The Strengths and Limitations of our Study

The strengths of this study lie in its naturalistic design, which makes the findings highly relevant for clinical practice. Additionally, the study incorporates responses from numerous network meetings with diverse participants. Furthermore, our decision to group all network members together in the analysis allowed us to compare the patients’ experiences with those of network members from outside the treatment system. This comparison offers new insights into the specific needs and vulnerabilities of participants in dissimilar roles during network meetings. However, this way of stratifying the sample did not allow for the analysis and comparison of experiences across different positions within the social network.

One limitation of this study is the imbalance in data quantity, with more written statements from network members than patients, as the network members formed a larger group. However, our more profound understanding of the patients’ context helped balance the difference in data richness between the two groups. Another limitation is related to the nature and analysis of the data, which consisted of relatively short written statements coded in the middle range between semantic and latent interpretation. Interpretive analyses are susceptible to confirming assumptions, but the involvement of three researchers with diverse perspectives and varying degrees of proximity to the data helped enhance the trustworthiness of the reflective work. Furthermore, the fact that our analyses yielded surprising results also supports the dependability of the findings. For example, we did not expect the uncertainty among network members to be as evident as it was, raising the possibility that the anonymous answers obtained immediately after meeting participation provided access to spontaneous emotional states that may otherwise have been missed. A final limitation is the measurement of fidelity, which was indirectly assessed by gathering responses from the respondents about their meeting experiences rather than by conducting more systematic evaluations.

### Implications for Future Research

This research points to the importance of exploring different participant roles in network meetings, such as parents, partners, siblings, and friends, to develop a deeper understanding of their unique perspectives. It also appears essential to further examine the meeting leader’s role in various treatment contexts. Moreover, although patients and network members in this study responded positively to the network meetings, we lack knowledge about whether participation influenced the dialogue in their social network outside the treatment system and the relationship between dialogic changes and mental health. Thus, further research is needed to explore the broader impact of participation in network meetings.

## Conclusions

Participating in one or two dialogical network meetings at a mental health outpatient clinic was considered highly valuable by both patients and network members. By comparing their experiences, similarities were found in their emphasis on getting the opportunity to express themselves freely and on the meeting leaders’ role in facilitating discussions on important and difficult matters. Differences included the network members’ need for more predictability and the patients’ emphasis on the importance of feeling understood and enhancing trust within the relationships. Mattering appears as a fruitful conceptual framework for describing and understanding processes within network meetings.
